# Proteomic analysis of the tear film in patients with keratoconus

**Published:** 2010-10-13

**Authors:** Isabel Lema, David Brea, Raquel Rodríguez-González, Elío Díez-Feijoo, Tomás Sobrino

**Affiliations:** 1 Instituto Galego de Oftalmoloxía, Servizo Galego de Saúde, University of Santiago de Compostela, Santiago de Compostela, Spain; 2 Clinical Neuroscience Research Laboratory, Hospital Clínico Universitario, IDIS, University of Santiago de Compostela, Santiago de Compostela, Spain

## Abstract

**Purpose:**

To identify proteins differentially expressed between the tear film of keratoconus (KC) patients and control subjects using two dimensional electrophoresis (2-DE) and mass spectrometry-based techniques.

**Methods:**

Twenty two patients (44 eyes) diagnosed with bilateral KC and 22 control subjects (44 eyes) were studied in a prospective case-control study. Keratoconus screening programs and Orbscan II topographies were performed on all participants. Tear samples were collected by the Schirmer I method using filter paper. Proteins were extracted from the Schirmer strips and separated by 2-DE. Comparison of protein patterns was performed using PDQuest Software and protein differences were identified by mass spectrometry. Finally, results were validated by western-blot.

**Results:**

Four spots were identified to be differentially expressed between KC patients and control subjects. Three of them were more expressed in healthy subjects and they were identified as zinc-α2-glycoprotein (ZAG), lactoferrin, and IGKC (immunoglobulin kappa chain). The other spot was more expressed in KC patients and it was identified as ZAG. Differences in ZAG seem controversial in two different spots because different posttranslational modifications, however, analysis of both spots revealed that globally, ZAG is overexpressed in healthy subjects. Founded differences in ZAG, lactoferrin, and IGKC expression were subsequently validated by western blot.

**Conclusions:**

IGKC protein, ZAG, and lactoferrin are under-expressed in the tears of patients diagnosed with bilateral KC compared with healthy subjects. These differences could contribute to the knowledge of the pathophysiology of this disease.

## Introduction

Keratoconus (KC) is a primary corneal ectasia, generally bilateral and progressive, with a conical shape as a result of the thinning of the stroma. This thinning induces irregular astigmatism, myopia, and protusion, leading to mild to marked impairment in the quality of vision [1]. KC has its onset at puberty, and it progresses until the third or fourth decade of life. The calculated incidence of KC is between 1/500 and 1/2,000 in the general population [1,2]. KC affects young persons in a very productive period of their lives. Yet the cause of this disorder, considered to be the most common indication for penetrating keratoplasty in developed countries [3], remains unknown.

KC may be isolated or may be associated with various clinical conditions, or even form part of a syndrome. Its etiology remains poorly understood, but it has been reported to arise as a consequence of biochemical alterations to corneal collagenase, which breaks down collagen leading to stromal thinning [4]. Other authors have suggested that it may arise as a consequence of keratocyte loss [5], which is on average about 19% lower in contact lens users [6]. In some atopic diseases, KC may be due to apoptosis of corneal cells, possibly mediated by interleukin-1 [7]. Rahí et al. [8], found atopic diseases in 35% of patients with KC, in contrast to 12% of control subjects. KC is frequently associated with ocular itchiness and rubbing, provoking chronic epithelial lesions [7]. The cornea is part of an integrated system - the ocular surface - which contains specific and non-specific immune molecules. Tissue degradation in thinning disorders, such as KC, involves the expression of inflammatory mediators, such as proinflammatory cytokines, cell adhesion molecules, and matrix metalloproteinases [9-12]. Moreover, previous studies from our group have demonstrated that the levels of proinflammatory cytokines (interleukin-6 [IL-6] and the tumor necrosis factor alpha [TNF-α]) and matrix metalloproteinase-9 (MMP-9) are significantly increased in the tears of patients with KC [13]. Despite these advances, there are currently no diagnostic markers of KC and the pathophysiology of this disease is largely unknown.

Proteomics is a discipline that study complex mixtures of proteins. Two-dimensional electrophoresis (2-DE) and mass spectrometry are very useful techniques to find out protein differences between different types of samples. Tear proteomics has been used to find out potential cancer markers [14], to study responses in patients with fungal keratitis [15] and for identification of tear proteases [16].

In this study we used proteomic tools, such as two dimensional electrophoresis (2-DE) and mass spectrometry, to find out protein differences in tear samples that allow the differentiation between eyes of KC patients and normal controls.

## Methods

### Subjects and clinical examination

We have designed a prospective, case-controlled study in which 22 bilateral KC patients (44 eyes) and 22 normal control subjects (44 eyes) were recruited from the Contact lens Unit, Instituto Galego de Oftalmoloxía, Complexo Hospitalario Universitario de Santiago de Compostela, Spain. KC patients (50.0% male; mean age, 31.3±7.8 years) and control subjects (54.5% male; mean age, 30.8±7.6 years) had not worn contact lenses for a week prior the study. All patients were expressly cited for the purposes of this study, and all examinations were performed by the same researcher between September 2007 and April 2008. Data collected included gender, age, patient’s ocular history (eye rubbing), medical history (atopy), and family history of KC. This research was performed in accordance with the Declaration of Helsinki of the World Medical Association (2000). Protocol was approved by the Hospital's Research Ethics Committee, and informed consent was given by all patients and control subjects.

The biomicroscopy exam was performed to detect signs of KC and the corneal topography study was performed to quantify topographic parameters. The modified Rabinowitz-McDonnell test was used to confirm the diagnosis of KC, and the severity of KC was classified according to the steepest simulated keratometry reading on the keratometric map (K_2_ <45 D, mild; K_2_ between 45 and 52 D, moderate; K_2_ >52 D, severe KC) [17].

Inclusion criteria were; 1) Bilateral KC patients, severe in both eyes or severe and moderate, with biomicroscopic signs in both eyes. 2) Subjects in the control group with normal corneal topography maps.

Exclusion criteria included; 1) Previous surgical intervention in the anterior segment, or childhood corneal trauma or disease. 2) Existence of active or systemic inflammation, or ocular disease, or current treatment with systemic or local anti-inflammatory drugs.

### Instrumental

Basic examination instruments were a Topcon biomicroscope and Topcon refraction column (Topcon, Essebaan, Netherlands). As specific examination instruments we used a Keratron Scout (Opticon 2000; SpA, Rome, Italy) and an Orbscan II corneal topographer (Orbtek, Salt Lake City, UT).

Two-dimensional electrophoresis was performed in IPGphor (Bio-Rad, Hercules, CA) for the first dimension and ETTAN-DALTSIX for second dimension (GE, Uppsala, Sweden). Western blot was performed using Miniprotean 3 system and Trans blot SD semi-dry transfer cell (Bio-Rad). Gels and blots were digitized in FX-Proplus (Bio-Rad). Finally, MALDI-TOF mass spectrometer (MS) analyses were performed using a 4700 Proteomics Analyzer MALDI TOF/TOF MS (Applied Biosystems, Carlsbad, CA).

### Tear analysis

Tear samples were obtained by placing a Schirmer strip over the lower lip, approximately 3 mm from the lateral canthus, without previous instillation of drugs or vital dyes. The subject was instructed to close the eyes for the 5 min test duration, the wet length was not recorded but was observed to be within normal ranges in all cases (>10 mm). We collected four Schirmer strip for subject; samples were frozen and stored at −20 °C immediately. Proteins were extracted from the Schirmer strips by incubating with 200 μl of 100 mM ammonium bicarbonate at room temperature for 1 h. Then 600 μl cold acetone were added and incubated for 2 h at −20 °C. Precipitation of proteins was performed by centrifugation at 18,000× g. Precipitated proteins were resuspended in solubilization buffer (9 M urea, 2% CHAPS). Proteins were quantified by modified Bradford assay using BSA as protein standard.

### Two-dimensional electrophoresis

Two replicate gels were performed for 12 patients (24 eyes) and 12 control subjects (24 eyes). Protein (20 μg) was applied on 17 cm 3–10NL IPG strips and first dimension was performed until 45 kV h was achieved. Second dimension of gel electrophoresis was performed on 12.5% polyacrilamide gels on ETTAN-DALTSIX (GE) at 5 W per gel for 15 min, and then at 17 W per gel until the dye front reached the bottom of the gel. Analytical gels were stained with Flamingo Fluorescent Stain and visualized on Molecular Imager FX Pro-plus (Bio-Rad). Preparative gels were stained in 0.1% Coomassie Blue Brilliant R-250.

Gels were digitalized using a Bio-Rad Molecular Imager FX Pro-plus. Protein patterns were analyzed using PDQuest 7.0 from Bio-Rad. Gel spots of interest were manually excised from preparative gels and digested according to Shevchenko et al. [18].

MALDI-TOF MS analyses were performed. The obtained peptide mass fingerprints were used for protein identification. The non-redundant NCBI or SwissProt database was searched using MASCOT 1.9 through the Global Protein Server v3.5 from Applied Biosystems. All determinations were performed without knowledge of the corresponding clinical data (blind test).

### Western blot

Tears collected from 10 patients (20 eyes) with KC and 10 control subjects (20 eyes) were used to subsequent validation of expression differences found using proteomics. Ten μg of total protein extracted from the Schirmer strips were loaded and size-separated in a 10% sodium dodecyl sulfate-polyacrilamide gel electrophoresis (95 V). The proteins were blotted in semi-dry conditions using polyvinyl difluoride membranes (Millipore, Billerica, MA) and incubated with primary antibodies raised in mouse against lactoferrin (1:2,000; Abcam, Cambrigde, UK), immunoglobulin kappa constant (IGKC) (1:2,000; Abcam) and zinc-α2-glycoprotein (1:700; Santa Cruz Biotechnology, Santa Cruz, CA) overnight at 4 °C. For detection, a goat anti-mouse Cy3-labeled secondary antibody (1:3,000; GE) was used and images were acquired using Molecular Imager FX Pro-plus (BioRad). QuantityOne software (BioRad) was used to analyze the expression of the different bands of protein. To verify equal protein loading, membranes were stripped at 50 °C for 30 min in stripping buffer containing 62.5 mM Tris, 2% SDS and 100 mM β-ME, subsequently re-blocked and probed with glyceraldehyde-3-phosphate dehydrogenase (GAPDH) antibody (1:3,000; Abcam). Experiments were performed in duplicate.

### Statistical analysis

Descriptive statistical analyses were performed with percentage for categorical variables. Discontinuous variables were expressed as median [quartiles]. Statistical significance for intergroup differences was assessed by the χ2 test for categorical variables. One-way ANOVA and the Mann–Whitney test were performed for comparison between groups. A value of p<0.05 was considered to be statistically significant. On the other hand, protein patterns were analyzed using PDQuest 7.0 from Bio-Rad. The reproducibility of the gels was calculated by Pearson's coefficient. Differences were considered when spot intensity was significantly different between patients and controls (p<0.01) and at least two times greater in one group respect to the other. The statistical analysis was conducted using SPSS 16.0 (IBM, Chicago IL) for Windows XP (Microsoft Corporation, Redmond, WA).

## Results

### Clinical Features

Table 1 shows clinical characteristics of patients with keratoconus and control subjects. No age-related or sex-related statistical differences were detected between KC patients and control subjects. A family history of KC was reported for 3 patients (13.6%) and 1 control (4.5%). Ten KC patients (45.5%) and five controls (22.7%) were diagnosed as having atopic disease (most commonly to pollen or acaroids). Twelve KC patients (54.5%) admitted frequent and vigorous eye rubbing and 3 control eye rubbing (13.6%).

**Table 1:** Clinical characteristics of patients with keratoconus and control subjects.

**Variables**	**Keratoconus (n=22)**	**Control (n=22)**	**p**
Age (years)	31.3±7.8	30.8±7.6	0.876
Males, n (%)	11 (50.0)	12 (54.5)	0.763
Atopic disease, n (%)	10 (45.5)	5 (22.7)	0.052
Eye rubbing, n (%)	12 (54.5)	3 (13.6)	0.005
K2, diopters	54.8±7.1	43.7±1.6	<0.0001
Thinnest P (μm)	386.2±71.6	542±23.9	<0.0001

All of the KC eyes showed either moderate or severe stage of progression: 18 eyes (40.9%) presented moderate KC (K_2_ between 45 D and 52 D), 26 eyes (59.1%) had severe KC (K_2_ >52 D).

Mean K_2_ was 54.8±7.1 D in the KC eyes, versus 43.7±1.6 D in the control subjects (p<0.0001). Mean of the thinnest point pachymetry was 386.2±71.6 µm in the KC eyes, versus 542±23.9 µm in the control subjects eyes (p<0.0001).

### Proteomic analysis

**Figure 1:**
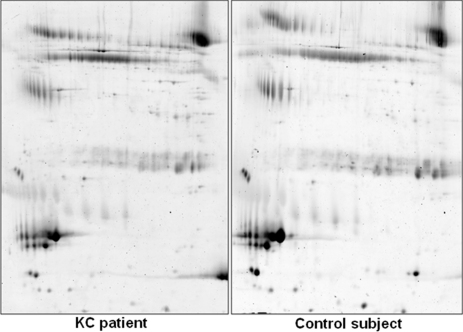
Master gel created by combining spots from all gels of keratoconus patients and control subjects.

**Figure 2:**
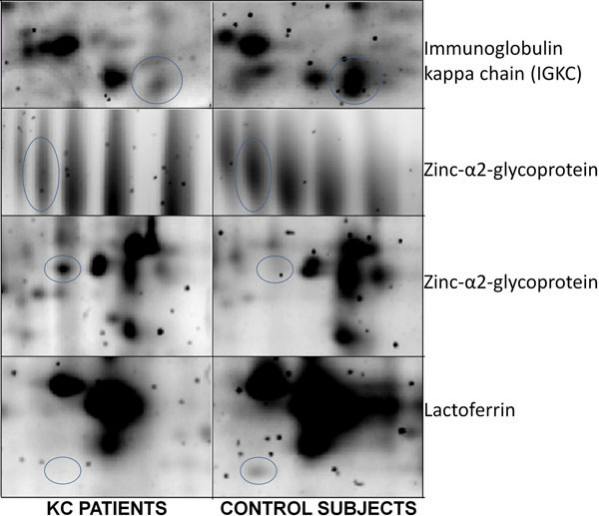
Two-dimensional gel fragments showing differential expressed proteins between keratoconus patients and control subjects. Three of them were more expressed in control subjects and they were identified as zinc-α2-glycoprotein (ZAG), lactoferrin, and IGKC (immunoglobulin kappa chain). The other spot was more expressed in KC patients and it was identified as ZAG.

**Figure 3:**
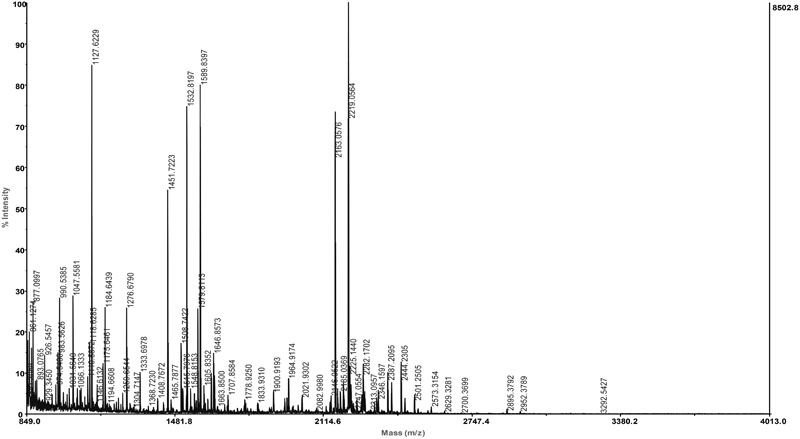
A typical profile of molecular mass of tryptic fragments of zinc- α2-glycoprotein (ZAG) during analysis by MALDI-TOF mass spectrometry.

An average of 532 spots was observed in both groups (Figure 1). Gels were highly reproducible (r^2^=0.95) between groups. No qualitative differences have been found, however three spots were found to be expressed at least twofold in healthy subjects than in KC patients and one spot was found twofold in KC patients than in healthy subjects (Figure 2). The expression levels are shown in Table 2. Furthermore these differences were statistically significant (p<0.001). Mass spectrometry analysis revealed that quantitative differences more expressed in healthy subjects were; IGKC (Immunoglobulin Kappa Chain) protein, zinc-α2-glycoprotein (ZAG; Figure 3), and lactoferrin and quantitative difference more expressed in KC patients was identified as ZAG.

**Table 2:** Protein expression values for the differentially expressed proteins between patients with keratoconus and control subjects. The values of the protein expression are showed as mean spot intensity (A.U.).

**Variables**	**Keratoconus (n=12)**	**Control (n=12)**	**p**
IGKC*	113.7±99.6	370.3±96.2	<0.001
ZAG†	819.1±247.2	2939.7±1700.9	<0.001
ZAG	63.5±23.4	16.8±2.9	<0.001
Lactoferrin	6.4±4.0	23.1±18.5	<0.001

*IGKC: immunoglobulin kappa chain. **†**ZAG: zinc-α2-glycoprotein.

### Western blot

**Figure 4:**
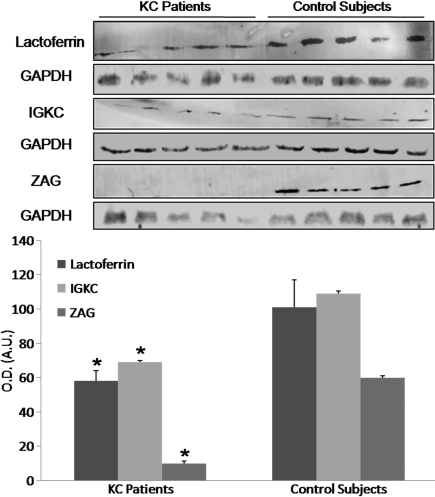
Western-blot analysis of the identified proteins showing a significant decrease in protein expression of zinc-α2-glycoprotein (ZAG), lactoferrin, and IGKC (immunoglobulin kappa chain) in KC patients compared to control subjects. O.D.: optical density; A.U.: arbitrary units; GAPDH: glyceraldehyde-3-phosphate dehydrogenase; *p<0.05 versus control subjects.

Western blot analysis for IGKC protein, ZAG, and lactoferrin showed a statistically significant decrease in protein expression (optic mean intensity) in KC patients compared to healthy controls (p<0.05; Figure 4).

## Discussion

Despite extensive basic and clinical studies of keratoconus in recent years, the precise mechanisms underlying this pathology still remain largely unknown. In this study, we performed a detailed proteomic analysis of both eyes in patients with bilateral KC and control subjects. Our results show the usefulness of proteomics to find out protein differences between KC patients and healthy subjects in tear samples. By comparing the global protein expression in tears, we found three spots whose expression was greater in the healthy subjects (IGKC protein, ZAG, and lactoferrin) and one spot was found to be more expressed in KC patients (ZAG). It is not strange to detect two or even more spots that belong to the same protein in a 2-DE gel. This is an indication that the protein has different posttranslational modifications. In our case, we have found two different spots for ZAG, one of them is threefold less expressed in KC patients respect to the controls, while the second spot is fourfold more expressed in patients than in control subjects. This is not a contradiction; one posttranslational modification could be more expressed in one group while the other could be just in the opposite way. Proteomics papers have described differences in posttranslational modifications in tears, specially homopolymers formation, protease degradation and glycosilation. Since ZAG is a glycoprotein, we hypothesized that posttranslational modification should be a glycosilation [19]. Nevertheless, what it is important is that overall the protein is more expressed in controls than in patients, as reflected in the western-blot results, where each band includes all posttranslational modifications (spots in 2-DE gels) of a protein. Therefore, we conclude that ZAG is under-expressed in KC patients respect to healthy subjects.

Tear lactoferrin is an 82 kDa protein produced in the acinar cells of the lacrimal gland. Lactoferrin is present in normal tears of humans and was found to be invariant through age and independent of sex. It has been reported reduced levels in certain diseases such as Sjögren syndrome, idiopathic dry eye, vernal conjunctivitis, contact lens induced giant papillary conjunctivitis, trachoma, herpes simplex keratitis, chronic irritative conjunctivitis keratoconjunctivitis sicca, and clinical dry eye with a marked keratopathy [20]. In tear film lactoferrin is thought to be antimicrobial and antiinflammatory. In particular, it has been demonstrated that Lipopolysaccharid*e*-induced production of IL-6, TNF-α, IL-1-β, and IL-8 is inhibited by lactoferrin, in various human monocytic cell lines [21-24]. Previous studies from our group have demonstrated that the levels of proinflammatory cytokines (interleukin-6 [IL-6] and the tumor necrosis factor alpha [TNF-α]) are significantly increased in the tears of patients with KC [13]. Likewise, it has been postulated that reactive oxygen and nitrogen species are raised in keratocunus that, in turn, induce collagenases and gelatinases responsible for the corneal thinning process in this disease [13,25]. Consequently, it is tempting to postulate that lactoferrin might be protective in keratoconus because of its role in decreasing the expression of proinflammatory cytokines and collagenases.

ZAG is a 41-kDa protein component of plasma, saliva, and tears. It stimulates lipid breakdown in adipocytes and is associated to the extreme weight loss that occurs in some cancers [26]. On the other hand, structural organization and fold is similar to MHC class I antigen-presenting molecule; hence, ZAG may have a role in the expression of the immune response [27]. We have demonstrated that the tears of patients with keratoconus contain a smaller amount of ZAG. Contradictory results were found in patients with Graves ophthalmopathy where the expression of ZAG is increased [28]. However, the specific role of ZAG in tears is not known.

Finally, we found a significant decrease in protein expression for IGKC in KC patients. IGKC and immunoglobulin kappa light chain variable region constitutes the light chains of an antibody and is related with immune response. In recent years, several clinical studies of KC support the idea that its pathogenesis involves an immune and inflammatory component [29,30]. Indeed, previous studies from our group, together with others, have shown that the levels of proinflammatory cytokines are significantly increased in the tears of patients with KC [10,13,31].

The limited sample size of the present study weakens the strength with which we may draw our conclusions. However, the robustness of the results gives support to the need to explore the role of these molecular markers as a new therapeutic or diagnostic tool in the KC. Although sample size is not so large, similar proteomic studies have used similar sample sizes [32]. Another limitation is that proteomics based on two-dimensional gels has a sensitivity in the order of magnitude of nanograms. Therefore, proteins whose expression is below the nanogram scale cannot be detected on the gels. Consequently, we do not rule out the possibility that other differences (not identified because of the sensitivity of staining methods) could exist between these two groups.

In conclusion, this study reveals that IGKC protein, zinc-α2-glycoprotein, and lactoferrin are under-expressed in the tears of patients diagnosed with bilateral KC compared with healthy subjects. However, the precise role of each of these molecular factors still needs to be defined, but the results suggest that the pathogenesis of KC might involve immune and inflammatory processes.

## References

[1] Krachmer JH Feder RS Belin MW Keratoconus and related noninflammatory corneal thinning disorders. Surv Ophthalmol 1984 28 293 322 6230745 10.1016/0039-6257(84)90094-8

[2] Rabinowitz YS Keratoconus. Surv Ophthalmol 1998 42 297 319 9493273 10.1016/s0039-6257(97)00119-7

[3] Dobbins KR Price FW Jr Whitson WE Trends in the indications for penetrating keratoplasty in the midwestern United States. Cornea 2000 19 813 6 11095055 10.1097/00003226-200011000-00010

[4] Tuft SJ Moodaley LC Gregory WM Davison CR Buckley RJ Prognostic factors for the progression of keratoconus. Ophthalmology 1994 101 439 47 8127564 10.1016/s0161-6420(94)31313-3

[5] Kaldawy RM Wagner J Ching S Seigel GM Evidence of apoptotic cell death in keratoconus. Cornea 2002 21 206 9 11862097 10.1097/00003226-200203000-00017

[6] Erie JC Patel SV McLaren JW Nau CB Hodge DO Bourne WM Keratocyte density in keratoconus. A confocal microscopy study(a). Am J Ophthalmol 2002 134 689 95 12429244 10.1016/s0002-9394(02)01698-7

[7] Wilson SE He YG Weng J Li Q McDowall AW Vital M Chwang EL Epithelial injury induces keratocyte apoptosis: hypothesized role for the interleukin-1 system in the modulation of corneal tissue organization and wound healing. Exp Eye Res 1996 62 325 7 8795451 10.1006/exer.1996.0038

[8] Rahi A Davies P Ruben M Lobascher D Menon J Keratoconus and coexisting atopic disease. Br J Ophthalmol 1977 61 761 4 603783 10.1136/bjo.61.12.761 PMC1043115

[9] Chandler JW Immunology of the ocular surface. Int Ophthalmol Clin 1985 25 13 23 3891657 10.1097/00004397-198502520-00004

[10] Bonini S Lambiase A Sgrulletta R Bonini S Allergic chronic inflammation of the ocular surface in vernal keratoconjunctivitis. Curr Opin Allergy Clin Immunol 2003 3 381 7 14501439 10.1097/00130832-200310000-00011

[11] Zhou L Sawaguchi S Twining SS Sugar J Feder RS Yue BY Expression of degradative enzymes and protease inhibitors in corneas with keratoconus. Invest Ophthalmol Vis Sci 1998 39 1117 24 9620070

[12] Collier SA Madigan MC Penfold PL Expression of membrane-type 1 matrix metalloproteinase (MT1-MMP) and MMP-2 in normal and keratoconus corneas. Curr Eye Res 2000 21 662 8 11148603

[13] Lema I Duran JA Inflammatory molecules in the tears of patients with keratoconus. Ophthalmology 2005 112 654 9 15808258 10.1016/j.ophtha.2004.11.050

[14] de Freitas Campos C Cole N Van Dyk D Walsh BJ Diakos P Almeida D Torrecilhas A Laus JL Willcox MD Proteomic analysis of dog tears for potential cancer markers. Res Vet Sci 2008 85 349 52 18164356 10.1016/j.rvsc.2007.11.006

[15] Ananthi S Chitra T Bini R Prajna NV Lalitha P Dharmalingam K Comparative analysis of the tear protein profile in mycotic keratitis patients. Mol Vis 2008 14 500 7 18385783 PMC2268856

[16] de Souza GA Godoy LM Mann M Identification of 491 proteins in the tear fluid proteome reveals a large number of proteases and protease inhibitors. Genome Biol 2006 7 R72 16901338 10.1186/gb-2006-7-8-r72 PMC1779605

[17] Zadnik K Barr JT Gordon MO Edrington TB Biomicroscopic signs and disease severity in keratoconus. Collaborative Longitudinal Evaluation of Keratoconus (CLEK) Study Group. Cornea 1996 15 139 46 8925661 10.1097/00003226-199603000-00006

[18] Shevchenko A Wilm M Vorm O Mann M Mass spectrometric sequencing of proteins from silver-stained polyacrylamide gels. Anal Chem 1996 68 850 8 8779443 10.1021/ac950914h

[19] Green-Church KB Nichols KK Kleinholz NM Zhang L Nichols JJ Investigation of the human tear film proteome using multiple proteomic approaches. Mol Vis 2008 14 456 70 18334958 PMC2268847

[20] Flanagan JL Willcox MD Role of lactoferrin in the tear film. Biochimie 2009 91 35 43 18718499 10.1016/j.biochi.2008.07.007

[21] Puddu P Valenti P Gessani S Immunomodulatory effects of lactoferrin on antigen presenting cells. Biochimie 2009 91 11 8 18539153 10.1016/j.biochi.2008.05.005

[22] Choe YH Lee SW Effect of lactoferrin on the production of tumor necrosis factor-alpha and nitric oxide. J Cell Biochem 1999 76 30 6 10580998 10.1002/(sici)1097-4644(20000101)76:1<30::aid-jcb4>3.3.co;2-l

[23] Mattsby-Baltzer I Roseanu A Motas C Elverfors J Engberg I Hanson LA Lactoferrin or a fragment thereof inhibits the endotoxin-induced interleukin-6 response in human monocytic cells. Pediatr Res 1996 40 257 62 8827774 10.1203/00006450-199608000-00011

[24] Håversen L Ohlsson BG Hahn-Zoric M Hanson LA Mattsby-Baltzer I Lactoferrin down-regulates the LPS-induced cytokine production in monocytic cells via NF-kappa B. Cell Immunol 2002 220 83 95 12657243 10.1016/s0008-8749(03)00006-6

[25] Shoham A Hadziahmetovic M Dunaief JL Mydlarski MB Schipper HM Oxidative stress in diseases of the human cornea. Free Radic Biol Med 2008 45 1047 55 18718524 10.1016/j.freeradbiomed.2008.07.021

[26] Russell ST Zimmerman TP Domin BA Tisdale MJ Induction of lipolysis in vitro and loss of body fat in vivo by zinc-alpha-(2)-glycoprotein. Biochim Biophys Acta 2004 1636 59 68 14984739 10.1016/j.bbalip.2003.12.004

[27] Hassan MI Waheed A Yadav S Singh TP Ahmad F Zinc alpha 2-glycoprotein: a multidisciplinary protein. Mol Cancer Res 2008 6 892 906 18567794 10.1158/1541-7786.MCR-07-2195

[28] Baker GR Morton M Rajapaska RS Bullock M Gullu S Mazzi B Ludgate M Altered tear composition in smokers and patients with Graves ophthalmopathy. Arch Ophthalmol 2006 124 1451 6 17030713 10.1001/archopht.124.10.1451

[29] Chandler JW. Ocular surface immunology. In: Pepose JS, Holland GN, Wilhelmus KR, editors. Ocular infection and immunity. St Louis: Mosby; 1996. p. 104–11.

[30] Bonini S, Lambiase A, Juhas T, Rama P. Inflammatory immune-associated diseases of the cornea. In: Ben Ezra D, editor. Ocular inflammation: basic and clinical concepts. London: Martin Dunitz; 1999. p. 151–68.

[31] Lema I Sobrino T Durán JA Brea D Díez-Feijoo E Subclinical keratoconus and inflammatory molecules from tears. Br J Ophthalmol 2009 93 820 4 19304583 10.1136/bjo.2008.144253

[32] Srivastava OP Chandrasekaran D Pfister RR Molecular changes in selected epithelial proteins in human keratoconus corneas compared to normal corneas. Mol Vis 2006 12 1615 25 17200661

